# The Art of the Special Interest: Lexicon-Based Analysis of Longitudinal Changes in Language Patterns Among Neurodiverse Youth Designing Special Interest–Related Digital Art

**DOI:** 10.2196/59976

**Published:** 2025-09-08

**Authors:** Celeste Campos-Castillo, Elise Atkinson, Uta Nishii

**Affiliations:** 1 Department of Media and Information Michigan State University East Lansing, MI United States; 2 Department of Statistics and Probability Michigan State University East Lansing, MI United States

**Keywords:** online learning, e-learning, virtual learning, text analysis, focused interests, circumscribed interests, repetitive behaviors, autism, ADHD, art therapy, special education, life skills, neurodiverse

## Abstract

We estimated linear mixed-effects models to analyze changes in language patterns (as measured using Linguistic Inquiry and Word Count) among neurodiverse youth to introduce a novel assessment useful for research into the potential benefits of special interests while minimizing respondent and researcher burden.

## Introduction

Special interests (SPINs) are circumscribed interests that may interfere with learning and socialization among neurodiverse youth [1-3]. Research has shifted to identify how SPINs can benefit neurodiverse youth [1,4-6] but relies on biased guardian or teacher reports [4,5]. We introduce a novel assessment that analyzes the speaking turns of neurodiverse youth during an observational study where they cocreated SPIN-related artwork with mentors. To characterize language patterns, we used Linguistic Inquiry and Word Count (LIWC-22, Pennebaker Conglomerates, Inc) [7], which has been used to automate the analysis of spoken words from neurodiverse populations [8,9]. Through examining how language patterns change when mentors engage SPINs, we demonstrate how SPINs may be advantageous while reducing respondent and researcher burden.

## Methods

### Ethical Considerations

The institutional review board of the University of Wisconsin-Milwaukee granted ethics approval (approval number 22.304). Written consent and assent were collected using a web-based form. Data were deidentified by assigning a pseudonym to participants. Participants were compensated with US $20.

### Overview

A convenience sample of neurodiverse youth whose traits were consistent with level 1 (requiring support) or 2 (requiring substantial support) autism diagnoses [10] participated in five weekly 75-minute Zoom (Zoom Communications) sessions [11]. Each session started with an icebreaker in the main room (5 minutes); the student-mentor dyads then entered breakout rooms to cocreate digital artwork using Blender (Blender Foundation), Photoshop (Adobe), or Procreate (Savage Interactive; 60 minutes); and finally everyone returned to the main room to screenshare artwork (10 minutes). Mentors were advised to engage their student’s SPIN to build rapport and brainstorm the artwork’s topic. Because SPIN engagement was not required, it produced variation.

A total of 9 neurodiverse youths participated (aged 10-25 years; sex: n=8 male, n=1 female; race: n=9 White). Each student was recorded at least once via Zoom, yielding 30 student sessions. The reasons for not recording were technical malfunction, absence, and lacking consent. The researcher turned off their video to minimize disturbance.

Multimedia Appendix 1 describes the transcript-processing to separate students’ speaking turns. Using LIWC-22, we examined word count and four linguistic categories: achievement (goal orientation), positive and negative emotion, and cognitive processing (active thinking). Scores for each category represent the percentage of words spoken that match words for that category (range 0-100). To determine whether the mentor engaged the student’s SPIN, the first two authors coded sessions independently (κ=0.83) and collectively made final determinations. The observed strategies were asking the student about the SPIN, asking the student to share other SPIN-related artwork, and participating in drawing SPIN-related artwork.

For each language pattern, we estimated two linear mixed-effects models to identify students’ language patterns over the five sessions and changes associated with engaging with SPINs. In both, the fixed effects were student identifiers (1-9) and session numbers (1-5); the random effects were student identifiers. The second models added a fixed effect for whether a mentor engaged the SPIN in the student-session (yes/no), which were interacted with the fixed effects for session numbers. Mentors always engaged the SPIN in the first session, creating collinearity, thus the models in the second session (n=26) exclude these. Stata 18.1 (StataCorp) was used for the analysis, with a statistical significance set at .05 (2-tailed).

## Results

The mean word count was 675.3 (SD 783.3). The means for achievement (1.1, SD 1.5), cognitive processing (10.0, SD 4.9), positive emotion (1.8, SD 3.3), and negative emotion (0.1, SD 0.3) were on the lower ranges. Table S1 in Multimedia Appendix 1 shows excerpts from student sessions that scored high and low.

**Figure 1:**
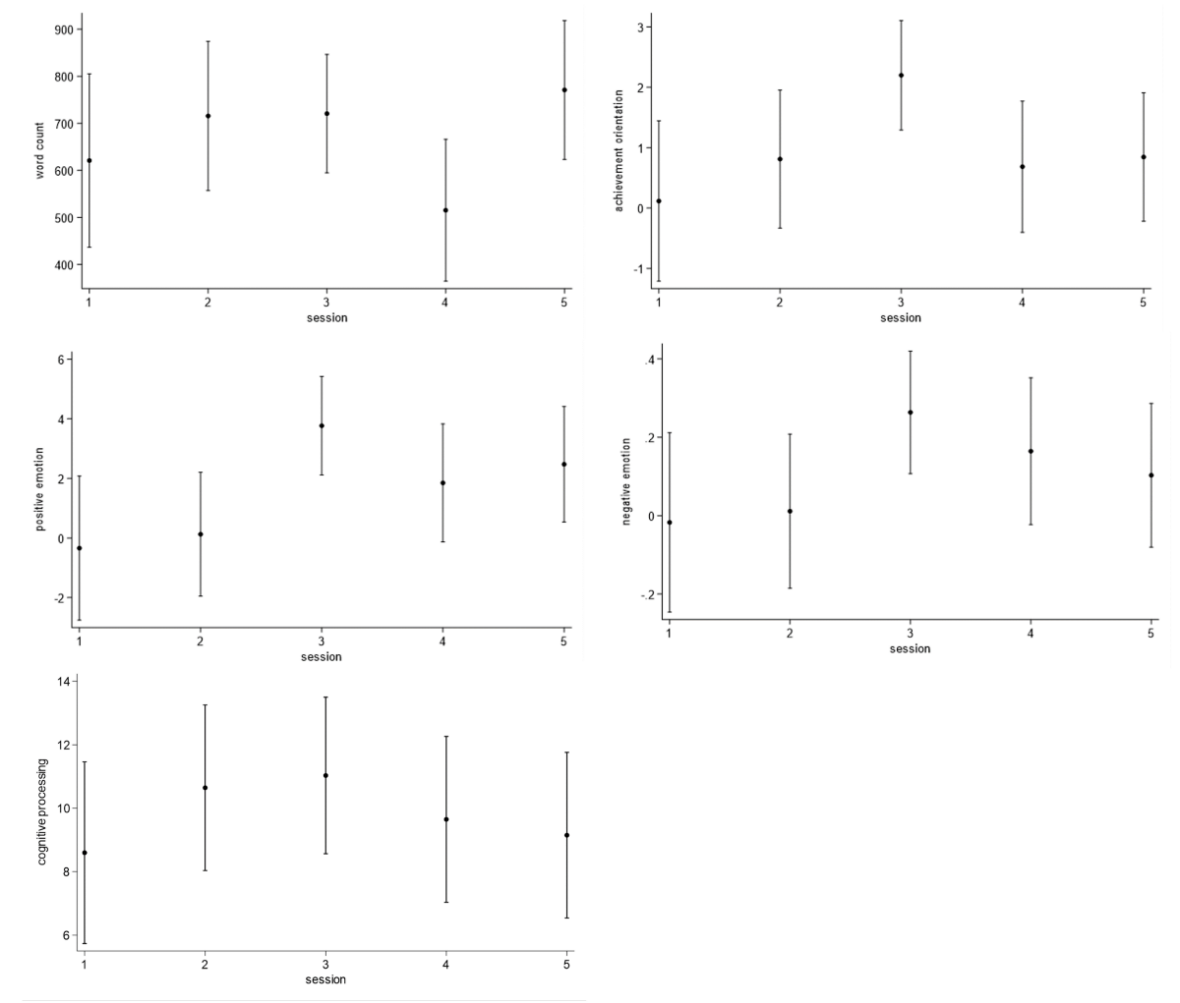
Predicted word count and category scores with 95% CIs by session number among neurodiverse youth in observational study.

**Figure 2:**
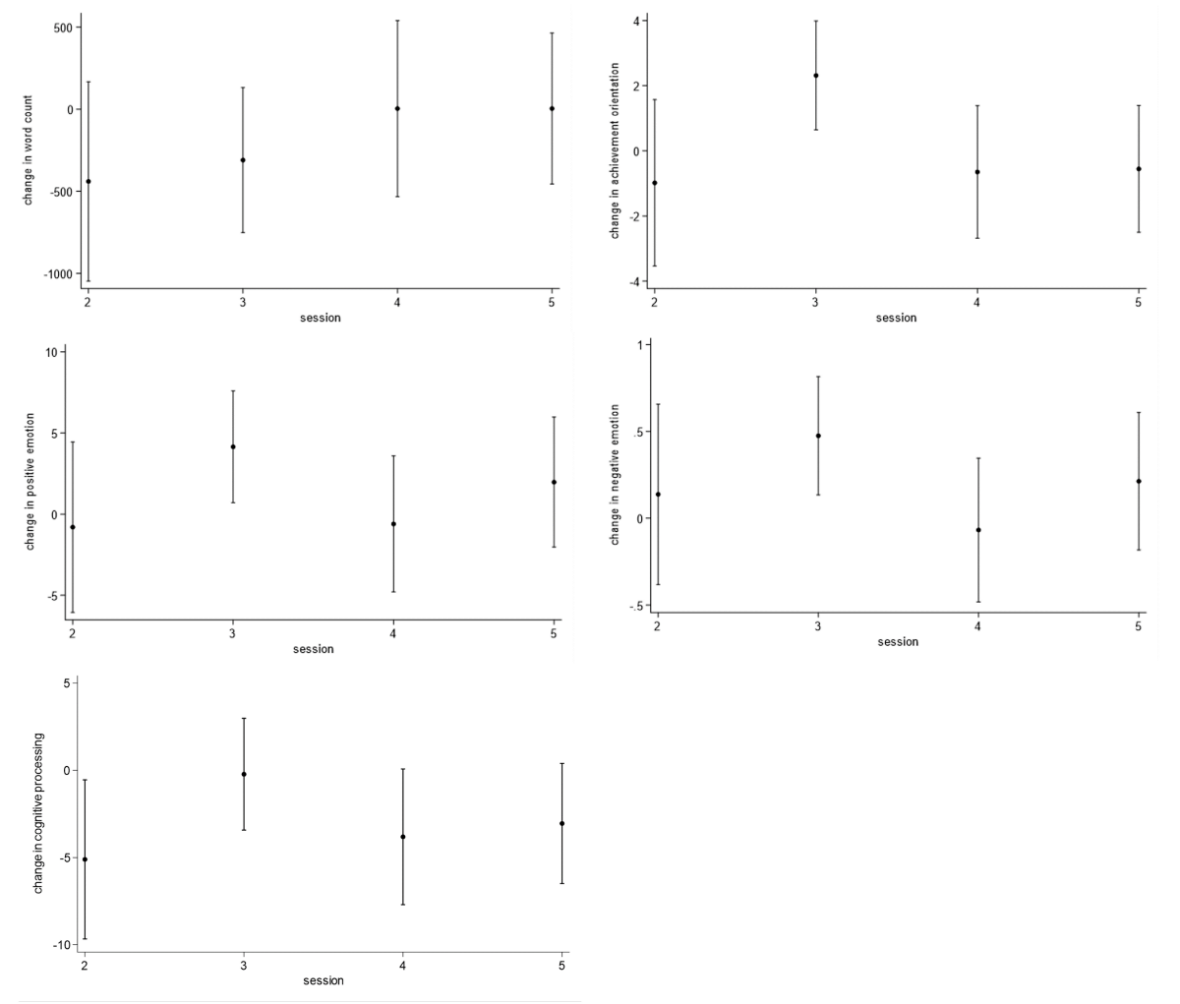
Changes (with 95% CI) in word count and category scores attributable to special interest engagement among neurodiverse youth in observational study.

Tables S2 and S3 in Multimedia Appendix 1 show coefficients for the linear mixed-effects models. Based on the first set of models, Figure 1 shows that scores, excluding word count, were significantly higher in the third session versus the first. Based on the second set of models, Figure 2 shows that engaging SPINs was associated with increased achievement and emotion (positive, negative) scores during the third session but decreased scores for cognitive processing in all but the third. Table S1 in Multimedia Appendix 1 shows excerpts from the third session, specifically “Gerardo” celebrating achievements with a song and “Kaya” expressing concern over a setback.

## Discussion

Words reflecting emotion and achievement were spoken more when mentors engaged with SPINs, which aligns with research indicating that SPINs can enhance social communication and learning but typically rely on guardian or teacher reports [4-6]. Using LIWC to analyze language patterns could reduce respondent and researcher burden while advancing research on SPINs.

Limitations include a small homogenous sample and being unable to compare long-term influences of different strategies to engage students’ SPINs (each mentor used only one). Perhaps asking about the SPIN enhances the word count (ie, speaking more) compared to other strategies. Future observational research with larger, more diverse samples would enable identifying differential impacts.

## Appendix

Multimedia Appendix 1 Description of transcript processing, excerpts from transcripts, and estimated coefficients in linear mixed-effects models.

## Abbreviations

SPIN: special interest

## Data Availability

Ethical restrictions limit making the videos and transcripts publicly available because participants did not consent to their videos being shared and only consented to have their transcripts shared after the final report to the National Endowment for the Arts. Requests to access deidentified transcripts may be sent to irbinfo@uwm.edu.
