# Supplementary material for: The Art of the Special Interest: Lexicon-Based Analysis of Longitudinal Changes in Language Patterns Among Neurodiverse Youth Designing Special Interest–Related Digital Art
Source: J Med Internet Res. 2025 Sep 8;27:e59976. doi: 10.2196/59976 (PMC12455143; doi:10.2196/59976)
Supplement: Multimedia Appendix 1 [file jmir_v27i1e59976_app1.docx]

**Multimedia Appendix**

**Description of Transcript Processing**

We used Microsoft to automatically transcribe student-sessions and manually checked for accuracy. The speaker identifiers for students and mentors were replaced with pseudonyms. A Python script was created to systematically extract and categorize text from students’ speaking-turns from the session transcripts. The script matched a search pattern (using the re module) to identify timestamps and speaker identifiers. The pattern, ^(\d{2}:\d{2}:\d{2})\s(.+), matches lines beginning with a timestamp followed by a speaker's identifier. For example, here is an excerpt from a student-session, specifically Pilar’s third session:

00:00:18 Mentor 4

Is did did it start rendering?

00:00:20 Mentor 4

I don't think it did.

00:00:20 Pilar

Yeah, it's it's rendering.

This approach facilitated the segregation of text by speaker and a unique text file was generated containing the entirety of a speaker’s statements during a session. File operations, conducted through the os module, involved creating an output directory and generating speaker-specific text files. For example, the script would take the above excerpt and generate two text files, the first with the following Mentor 4’s statements:

Is did did it start rendering?

I don't think it did.

The second text file would hold Pilar’s statements:

Yeah, it's it's rendering.

| Table 1. Sample excerpts from sessions scoring high and low on LIWC category scores among autistic youth | | |
| --- | --- | --- |
| LIWC Category | High | Low |
| Negative emotion | In his third session, Kaya experiences several computer problems. In one instance, he is attempting to undo a mistake he made, but is repeatedly unsuccessful: "Wait a minute. I need I need to. Fix this control Z control Z. No undo undo...No, something's wrong." | While attempting to log into his Dropbox during the first session, Gustavo tells the moderator, "Oh, hi [moderator]. I was just waiting for the login to pop up." |
| Positive emotion | Matthew’s statements in the third session included describing his past experiences with the special interests that comprised his digital art. For example, while drawing the character, Gumby, on Procreate, he shared with the mentor that he and his family had watched a movie about the character and exclaimed, “And the movie is great yes, we liked it.” | Pilar’s statements in the third session included describing a funny story from a YouTuber he watches with "Well, he calls himself Dani, but but everybody calls him Dani Milkman because he because he has this meme where he where he just, like, chugs an entire thing of milk. Like an entire carton." |
| Achievement orientation | Gerardo typically uses a letter board to communicate, however, when he did speak on his own, it was to celebrate his achievements. For example, at the end of the third session he sings, “Celebrate good times, come on.” | Early in Jayden's fifth session, his mentor asks if he has additional ideas to work on and he explains that he believes his artwork is complete: "No, that's basically it." |
| Cognitive processing | Taylor was learning to animate in Blender and struggled to make the eyes of his frog pop out. He explained his intentions to this mentor: “I actually wanted the, I actually wanted the body to, I just wanted the guy to open up his eyeballs. You know, like he's waking up.” | In the middle of drawing a Minecraft character, Zelda's mentor asks her what it will be. Zelda responds, "I'm trying to draw a frog. Some of the frogs have eyes that look like that." |
| Note: Pseudonyms are used to protect the confidentiality of research participants. | | |

| Table 2. Coefficients from mixed-effects models estimating word count and category scores among autistic youth | | | | | | | | | | | |
| --- | --- | --- | --- | --- | --- | --- | --- | --- | --- | --- | --- |
| Coefficient |  | Word Count | | Cognitive Processing | | Achievement Orientation | | Positive Emotion | | Negative Emotion | |
|  |  | b | p-value | b | p-value | b | p-value | b | p-value | b | p-value |
| Session (vs. 1) | | |  |  |  |  |  |  |  |  |  |
| 2 |  | 94.7 | 0.435 | 2.0 | 0.106 | 0.7 | 0.427 | 0.5 | 0.769 | 0.0 | 0.849 |
| 3 |  | 99.6 | 0.387 | 2.4 | 0.044 | 2.1 | 0.012 | 4.1 | 0.007 | 0.3 | 0.049 |
| 4 |  | -105.6 | 0.396 | 1.1 | 0.418 | 0.6 | 0.526 | 2.2 | 0.180 | 0.2 | 0.240 |
| 5 |  | 149.9 | 0.223 | 0.6 | 0.668 | 0.7 | 0.411 | 2.8 | 0.081 | 0.1 | 0.431 |
| Constant |  | 2098.3 | | 9.9 | | 0.1 | | -1.7 | | 0.1 | |
| N = 30 | | | | | | | | | | | |

| Table 3. Coefficients from mixed-effects models estimating changes in word count and category scores associated with SPIN engagement during student-sessions | | | | | | | | | | | |
| --- | --- | --- | --- | --- | --- | --- | --- | --- | --- | --- | --- |
| Coefficient |  | Word Count | | Cognitive Processing | | Achievement Orientation | | Positive Emotion | | Negative Emotion | |
|  |  | b | p-value | b | p-value | b | p-value | b | p-value | b | p-value |
| Session (vs. 2) |  |  |  |  |  |  |  |  |  |  |  |
| 3 |  | -180.7 | 0.489 | -3.2 | 0.126 | -0.7 | 0.614 | 0.1 | 0.966 | 0.0 | 0.911 |
| 4 |  | -573.2 | 0.064 | -2.4 | 0.326 | -0.6 | 0.686 | 0.7 | 0.823 | 0.3 | 0.396 |
| 5 |  | 307.7 | 0.239 | -3.8 | 0.065 | 0.6 | 0.680 | 0.0 | 0.998 | 0.0 | 1.000 |
| SPIN-engagement |  | -439.7 | 0.156 | -5.1 | 0.028 | -1.0 | 0.450 | 0.8 | 0.765 | 0.1 | 0.603 |
| Session * SPIN-engagement |  |  |  |  |  |  |  |  |  |  |  |
| 3 |  | 130.0 | 0.673 | 4.9 | 0.045 | 3.3 | 0.033 | 4.9 | 0.119 | 0.3 | 0.283 |
| 4 |  | 444.0 | 0.201 | 1.3 | 0.634 | 0.3 | 0.840 | 0.2 | 0.954 | -0.2 | 0.541 |
| 5 |  | 444.0 | 0.143 | 2.1 | 0.395 | 0.4 | 0.791 | 2.8 | 0.405 | 0.1 | 0.819 |
| Constant |  | 1128.0 | | 15.6 | | 1.49 | | 0.6 | | 0.0 | |
| N = 26 | | | | | | | | | | | |
